# Experiences of older adult Filipino-Americans surrounding eye surgery and factors in health decision-making: a qualitative study

**DOI:** 10.1186/s12913-024-12061-1

**Published:** 2024-12-18

**Authors:** Marycon C. Jiro, Michael Sigua, Migel Dio, Lauren Hennein, Jennifer Cocohoba

**Affiliations:** 1 University of California, San Francisco, Department of Medicine, San Francisco, CA USA; 2 University of California, Davis, Department of Medicine, Sacramento, USA; 3 University of California, San Diego, Department of Ophthalmology, San Diego, CA USA; 4 University of California, San Francisco, Department of Clinical Pharmacy, San Francisco, CA USA

**Keywords:** Qualitative, Interviews, Cataract, Minority, Filipino, Cultural humility, Patient-centered

## Abstract

**Background:**

The greater San Francisco metropolitan bay area is home to 270,000 Filipino immigrants and the second largest Filipino-American population in the United States. Despite this, Filipino-Americans are aggregated with the general “Asian-American” category, making it a challenge to obtain accurate population health data on social determinants of health. One area that is concerning is the lack of research on Filipino-American eye health experiences. The Filipino-American population is an older community with a median age of 48 years old that experiences a high prevalence of diabetes and hypertension. Preserving sight in high risk patients against age-related eye disease depends on routine eye examinations and timely treatment. Therefore, it is important to explore older adult Filipino-American eye surgery experiences and factors in eye health decision-making.

**Methods:**

An exploratory qualitative study was conducted with thirteen Filipino-American adults residing in the nine San Francisco Bay Area counties who had a history of eye surgery (cataract, glaucoma, or retinal). Data were collected through semi-structured in-depth interviews. A thematic analysis was performed informed by critical race theory and PEN3 cultural model. Standard methods of coding followed by determination of themes were conducted and consensus was reached among the three coders.

**Results:**

The mean participant age was 73.3 years old (95% CI 69.7–76.9). All participants were born in the Philippines, and 85% identified as female. Four themes emerged: *the value of social networks and self-efficacy in decision-making; the importance of informed communication; the integral role of trust and the physician–patient relationship; and the impact of cultural humility, beliefs, and concordance on ocular care.* Communication with trusted peers, clinicians and clinical staff prior to surgery was a key factor in alleviating worries and misconceptions, yet interpreter services were inconsistently offered. Many saw clinicians as experts, which dampened their agency in asking questions or elicited shame in obtaining a professional second opinion.

**Conclusions:**

How clinicians convey information is crucial. Filipino-Americans patients can interpret eye surgery expectations through a cultural lens, which may result in lasting impressions about the surgical experience and outcomes. Expanding cultural humility training and improved incorporating interpreter services can help patients feel supported.

**Supplementary Information:**

The online version contains supplementary material available at 10.1186/s12913-024-12061-1.

## Introduction

Visual impairment compromises activities of daily living, reduces social engagement, increases fall rates, and decreases quality of life [1–4]. According to a 2021 meta-analysis of several United States (U.S.) cohort studies, approximately 12 million people aged 40 years and older have some form of vision impairment, including one million who are blind and eight million who have uncorrected refractive errors [5, 6]. As the U.S. population continues to age, more individuals will experience some form of visual defect, making this a significant public health burden.

Filipino-Americans comprise the largest Southeast Asian and third largest Asian-American population in the U.S., with a median age of 51 years among foreign-born Filipinos and 44 years among those born in the U.S. [7]. However, there is markedly limited literature assessing the state of ophthalmic care in this minority group. This is of concern because risk factors that disproportionately affect Filipino-Americans, such as high blood pressure, diabetes, and structural inequities, [8, 9] place this population at increased risk of worsened eye health. Three studies conducted at community-based comprehensive, private ophthalmology clinics in northern California demonstrated that the prevalence of all forms of diabetic retinopathy (DR) among Filipinos was approximately twice as high when compared to the prevalence among Caucasians and that Filipino-American patients had a significantly greater prevalence of narrow anterior chamber angles of the eye, a risk factor for glaucoma [10–12]. Our recent study showed that while a large proportion of older Filipino-American adults in San Francisco are receiving adequate eye care, a significant gap in knowledge about eye health and diseases exist [13].

Eye surgeries, such as glaucoma, retinal, and cataract surgery, have been reported to improve quality of life and reduce depression, anxiety, the risks of falls and the limitation of activities [1, 3, 14]. Often effective at slowing down progression of eye disease, glaucoma surgery can require close postoperative monitoring and are associated with complications such as leakage, infection, failure, and long-term ocular surface irritation [14]. Ensuring surgical success remains paramount and can be achieved through different means such as preoperative patient education and detailed follow-up plans [15, 16]. Qualitative experiences before, during, and after surgery can provide insight into the factors that contribute to surgical success, which may not be observed from quantitative studies alone [17]. We conducted semi-structured, in-depth interviews with Filipino-Americans who have undergone cataract, retinal, or glaucoma surgery within the nine San Francisco (SF) Bay Area counties to gain insight into this older and expanding group’s ophthalmic health decision-making process and experience in obtaining surgical care. Our aims were to describe the expectations and beliefs of Filipino-Americans surrounding eye surgery; to understand how cultural beliefs, practices and support systems encourage or discourage eye care engagement; and to highlight participant-identified areas that can change the surgical care experience.

## Methods

The ethics committee of the University of California, San Francisco’s Human Research Protection Program and the University of California, Berkeley’s Office for the Protection of Human Subjects reviewed and deemed this study exempt human subjects research. This study complied with the Health Insurance Portability and Accountability Act and followed the tenets of the Declaration of Helsinki.

### Study design, inclusion criteria, and recruitment

Our inclusion criteria were self-identified Filipino-American individuals aged 40 and older who underwent eye surgery (cataract, retinal, or glaucoma), spoke English and/or Tagalog, and resided in one of the nine SF Bay Area counties (SF, San Mateo, Alameda, Contra Costa, Santa Clara, Marin, Napa, Sonoma, and Solano). We excluded eye surgery procedures performed outside of the U.S. Of note, the greater SF metropolitan area is home to the second largest Filipino population in the U.S. [7].

Over a 12-month period from June 2021 through June 2022, participants were recruited in-person through flyers and sign-up sheets at seven Filipino-American community events, organizations, and churches. We partnered with various Filipino-serving community organizations in the SF Bay that circulated our study via word of mouth and virtually through email. Participants in a previous research study on eye health who expressed interest in participating in future studies and met eligibility criteria were also contacted [13].

### Data collection and measures

The semi-structured interview guide was developed with consultation from an advisory group of healthcare professionals, including a pharmacist, ophthalmologists, master’s colleagues, and public health professors. Each semi-structured interview was meant to elicit information from participants regarding (1) general eye care, including their relationship with their provider; (2) the entirety of their experience with their eye surgery, involving their expectations on the procedure; (3) the COVID-19 pandemic and how it has affected their access to eye care; and (4) relationships with their community and their perspectives on eye care (Supplemental 1).

A nonprobabilistic purposive sampling strategy was used to document common patterns that cut across variations in participants. Interested participants were contacted via phone calls and screened for eligibility with standardized questions (Supplemental 2). Participants were recruited for in-depth, semi-structured telephone interviews conducted in either English or Tagalog based on the participant’s preference. Tagalog interviews were led by research team members fluent in the language. Interviews lasted between 60 and 90 min and were digitally audio-recorded. Informed consent was obtained from participants prior to recording the calls. These calls were transcribed and, if in Tagalog, translated verbatim by the research team. Following each interview, field notes were immediately written for each participant. A $20 gift card was emailed or mailed to those who completed the study.

### Data analysis

A systematic content analysis was conducted using both theory-driven and inductive approaches. An initial codebook was developed based on concepts from the PEN3 Cultural Model and Critical Race Theory (CRT). The PEN3 cultural model is a three-dimensional framework that addresses risk behaviors, protective factors and the roles each play in health and decision making by centering culture in the discussion about social determinants of health and health promotion [18]. CRT was incorporated to identify “counternarratives” in the participants’ lived experiences as people with minoritized identities and to explore how cultural norms, institutional practices and structural racism influence the health care decisions and outcomes of this population in eye care and surgery [19–21]. Each interview was manually coded independently by three researchers (MJ, MD, MS) on Microsoft® Excel Version 16.89.1 based on the initial codebook. The coding team met regularly to align, consolidate, and clarify codes to better categorize the responses that emerged from the interviews. Additional codes that consistently emerged from participants’ perspectives were incorporated into later versions of the codebook. After multiple iterations, a final set of codes was applied to all interviews. Data collection was ceased at 13 interviews because thematic saturation was reached; no new codes or themes emerged from the data.

## Results

### Demographics

A total of 13 semi-structured interviews were conducted, six in English and seven in Tagalog. The average age of the participants was 73.3 years old (95% CI 69.7–76.9)​. All participants were born in the Philippines, and 85% identified as female​. Most individuals had undergone cataract surgery (79%). Twelve out of thirteen participants reported an annual household income of at or below $60,000 (Table 1).

**Table 1:** Characteristics of Filipino-American participants who received eye surgery

Characteristic	Mean or N (*n* = 13)	%
Age, years	**73.3**	
Sex		
*Male*	**2**	**15**
*Female*	**11**	**85**
Country of Residence		
*Alameda*	**4**	**31**
*Antioch*	**1**	**8**
*Contra Costa*	**1**	**8**
*San Francisco*	**6**	**46**
*San Mateo*	**1**	**8**
Type of Surgery (n = 14)^a^		
*Cataract*	**11**	**79**
*Retina*	**1**	**7**
*Glaucoma*	**2**	**14**
Year of Most Recent Surgery		
*Before 2010*	**1**	**8**
*2010 – 2013*	**2**	**15**
*2014 – 2017*	**6**	**46**
*2018 – 2021*	**4**	**31**
Country of Birth		
*Philippines*	**13**	**100**
Interview Language		
*English*	**6**	**46**
*Tagalog*	**7**	**53**
Annual Income		
≤ *$60,000*	**12**	**92**
*$60,000–$100,000*	**1**	**8**
Highest Level of Education		
*Elementary*	**2**	**15**
*High School*	**1**	**8**
*Two Year College*	**1**	**8**
*Four Year College*	**6**	**46**
> *Four Year College*	**3**	**21**
# Household Individuals		
*Average*	**3**	
*Range*	**1–7**	
Primary Health Insurance		
*Government Sponsored*	**5**	**38**
*Private*	**7**	**54**
*None*	**1**	**8**
Has a Primary Care Provider		
*Yes*	**12**	**92**
*No*	**1**	**8**
Has an Optometrist		
*Yes*	**10**	**77**
*No*	**3**	**23**

^a^*One participant underwent both cataract and glaucoma surgeries*

### Emergent themes

Four major themes emerged from the interviews. These included *1) the value of social networks and self-efficacy in decision-making, 2) the importance of informed communication, 3) the integral role of trust and the physician–patient relationship,* and *4) the impact of cultural humility, beliefs, and concordance on ocular care* (Table 2).

**Table 2:** Emergent themes and select quotes from interviews regarding eye surgery among San Francisco Bay Area Filipino-Americans

Themes	Representative Quotes
***Social networks and self-efficacy in decision making***	
*Family and friends were often consulted prior to eye surgery. They would often advise the participants to be proactive and ask the clinician as many questions as possible; those that were not able to do so expressed regret in not being well-informed*	I just decided with my sister “come, come” ‘cause she's a [licensed vocational nurse (LVN)], “And you come and help me.” So, what happens if you don't have an LVN and you don't have anybody else with you at home. How are you going to do that? *– 77F, cataract*
	I asked, “May you wait doctor? May I ask my daughter? She’s in the office now; may I call her for a moment?” Then I told my daughter, and then my daughter approved. So, we, the doctor, did it. *– 74F, cataract*
	*[For eye information]* I usually would call the doctor or sometimes [use] the iPhone. They have a lot of things in there when you type in eye diseases… I'm my friends' big advisors… Haha. I don't mind about it if I can help someone. *– 75F, cataract*
	Make sure that you ask questions. I always tell friends, “Ask questions to your doctor. You know if you don't understand it.” So, that they have a better experience than I did. *– 77F, cataract*
	If you don’t say something they’re not going to help you. It’s not just you that they’re taking care of. For example, if you’re admitted into the hospital… if you’re not gonna say anything, the nurse will leave you alone. *– 75F, cataract*
***Importance of informed communication***	
*The explanation of the surgical process was associated with a participant’s positive or negative care experience. Some wished for an interpreter. Others did not need it but acknowledged how it could help in quickly and confidently sharing thoughts and expressions*	I was afraid that it would hurt… I remember years ago when I had heard from friends in the Philippines or somebody who had got the surgery. I always thought they would lay [me] down and… they would scrape [my eyes], that was my imagination. But when it was my turn they put on anesthesia, and it was just easy, it just went "*crack crack crack crack"* and it was okay. *– 77F, cataract*
	My surgery experience was excellent for me because everything was explained to me. I knew what I was expecting and the good rapport with my doctor and the nurses helped me out and all those explanations would calm you down and you would know what you expect. So, you won't be scared… First, I was sort of afraid to have it done but after all this preparation and everything… I felt comfortable. *– 75F, cataract*
	They just read me something, the permit… they had me sign it. It was the permission to do the operation. But of course, I cannot read that 50% because I already could not see (*laughs)*. And so, I just asked them, what is this? “This is the condition.” Yes, then I just signed it. *– 70 M, retinal disease*
	When you say it in the vernacular, or the Tagalog language, it becomes more intimate in terms of your relationship and the frankness and the honesty and what is needed. The doctor can calm you down, and you can talk also in Tagalog. That’s good. I'm all for it. *– 69 M, cataract*
	I think… [an interpreter] wouldn't have made a difference. Maybe for others, who totally need a translator… It can possibly help. Because, for me, for example myself… I know how to speak English. It’s just that, sometimes when you’re speaking to someone white or whatever… It’s like you get a mental block. "What was I supposed to say again? How do I express this in English?" You kinda have to translate it in your thoughts first before telling them about it. *– 56F, cataract and glaucoma*
***Integral role of trust and the physician–patient relationship***	
*Many trusted their physicians as experts and valued warm, welcoming characteristics as well as their competency in their work. At times, this innate trust impeded them from obtaining a second opinion and ultimately caused them to lose trust when a complication emerged*	My sister who's the one that drives me to the hospital, that is also what they told me, ‘You did not have a second opinion, why did you do it? why did you go through with it? You did not even tell us.' I thought that I already knew about things. That's the mistake… 'You have a chance to go through or back out… These are my eyes. That's what [my other] doctor was telling me, “It's not for me, it's for you. Take a second advice. Those are your eyes, if you lose them, that's it.” It's that way, and it's true!… “Just trust me, follow me, listen to me and everything will be alright,” [the operating surgeon had said]. Well for me, I lost my trust in him. *– 70 M, retinal disease*
	Yeah, well you're in America. Don't be afraid because everybody studied scientifically and medically and they're all experts in their line of their profession so don't feel afraid. go grab that opportunity when you need a medical care, or you'll need a care. You go… the earlier the better. For physician examinations. *– 74F, cataract*
	When I was talking to her, she was able to answer my questions. You can feel that she cares about you, by talking and she is well experienced. You usually feel that. She is really good for me, and she cares for her patients. She is approachable, you can see she is humble, and she even taps you as a gesture since there wasn't COVID yet back then. Always smiling on her face. You can feel that she is really a good eye doctor. *– 71F, cataract*
	You should just go to a specialist, do not just readily go to another doctor who does not have experience, because they might be studying still, or practicing. *– 84F, glaucoma*
***Impact of cultural humility, beliefs, and concordance on ocular care***	
*Faith and faith communities were big sources of support for many. Participants often rejoiced at the idea of a Filipino-American doctor but acknowledged that others may think of “American” doctors more highly. Some cited the “hiya” phenomenon as impacting one’s ability to fully engage in their physician–patient relationship*	I was thinking: "I might end up blind” haha. but I wasn't telling [the ophthalmologist] that I was scared of getting blind. I kept it to myself. Well of course, they might say that I don’t have faith in them. I always prayed that: “Lord, please help me so that my old vision would come back.” I always prayed. I was finding assurance in God that my vision before would come back. Mercy of God it did come back! *– 80F, cataract*
	I've worked with senior Filipinos for many years and that's one of the things… the "hiya"… when you feel "hiya" you don't ask (questions) anymore… then there are also Filipinos that may be a bit high class and educated who say, "I can understand (English), I don't like a Filipino doctor"…—*77F*,* cataract*
	And I think part of the community really helped because then it just made me feel better. You can say that my prayer group is praying for my healing… They send it to me via Facebook, or messenger and they go praying for your healing. Having that kind of lightens the whole experience. *– 72F, cataract*
	*On being asked if they would have preferred a Filipino-identifying doctor:* Yes, because I can express freely, independently, and normally. When I say without fear of expressing myself. We Filipinos understand each other. Yeah, I feel more comfortable… the feeling of oneness. That's the feeling of nationalism. *– 74F, cataract*
	I mean, that would be nice! But I don't know of any [Filipino ophthalmologist]. *– 76F, cataract*
	Oh, I would choose the Filipino-American doctor. They can speak Tagalog, or they can speak English. But I also understand what they are saying so that is ok. *– 84F, glaucoma*

### Social networks and self-efficacy in decision making

Social support from family members and peers was consistently mentioned when discussing eye surgery. Participants relied on the opinions of their family and friends to gain confidence in their eyecare and surgery decisions. At times, surgery did not occur unless a family member or friend was consulted. Positive feelings toward surgery were reinforced by friends and family, especially those who may have been health professionals themselves. Some participants also noted their ability to influence others’ eye surgery decisions by sharing their own positive or negative surgical experience. Moreover, autonomy and self-efficacy were two important factors participants cited that helped them obtain better eyecare experiences. Those with a positive eye surgery experience reported that simply asking questions helped them feel reassured about their surgery. Being forward and speaking out to tell providers what they needed was a positive trait that most participants highlighted. Alternatively, some participants reported that diligence in performing their research had helped them make favorable eye surgery decisions.

### Importance of informed communication

Participants reported that communication with their ophthalmologists and nurses prior to surgery helped ease their worries and misconceptions. Three participants relied on informal interpreters, such as the Tagalog-speaking clinic staff, to better understand the physician’s orders and ask clarifying questions about medical terminology. During preoperative sessions, professional interpreters were inconsistently offered to our participants. Only two of the thirteen participants were explicitly offered interpreters during their eye surgery appointments. Of the two who were offered formal interpreters, one participant declined the service because she felt confident in her English proficiency. The patient who accepted an official interpreter felt reassured and well informed. Six participants reported that using a formal interpreter would have been preferred if offered, especially if linguistic concordance with their ophthalmologist was possible. Two participants felt it would not have made a difference. Some patients expressed discomfort when asked whether they would like to use an interpreter. One participant felt that an interpreter would only help those who were less proficient in English. However, this particular participant shared that they struggled to express her thoughts in English during her one of her clinic appointments, asking herself, “What was I supposed to say again? How do I express this in English [to my doctor]?”.

### Integral role of trust and the physician–patient relationship

Overall, several participants described a sense of deference to the expertise of their doctors. One participant noted that because the doctors are “American trained,” they have more confidence in their abilities. They followed physician’s orders because they believed that they were receiving the best care. Some qualities of a good physician that were outlined by the participants were “kind,” “thoughtful,” “warm,” and “inviting.” Most stated that the competence of the physician outweighed other characteristics. Physicians were mostly obtained by referral, although those who had prior experience in healthcare more willingly researched the best physician that fit their needs and requirements. Many people said they would have desired a second opinion, especially if the outcome was not as expected. Some experienced difficulty obtaining a professional second opinion, and others shared their regret of being too trusting of their physician and not discussing their surgery with other trusted individuals.

### Impact of cultural humility, beliefs, and concordance on ocular care

Several participants reported that their faith was an important factor that helped alleviate fear and provided reassurance prior to and during the surgery. Prayer groups also helped build their confidence leading up to the surgery and provided participants with a sense of community leading up to the procedure and postoperatively. Additionally, the cultural phenomenon of “hiya,” otherwise defined in the literature as the “painful emotion arising from a relationship with an authority figure or with society, inhibiting self-assertion in a situation which is perceived as dangerous to one's ego,” [22] was repeatedly mentioned as a factor that decreased open communication with the ophthalmologist. For example, one participant expressed, *“When you feel "hiya" you don't ask (questions) anymore.”* Cultural concordance with the ophthalmologist was also desired, but participants reported that this was not necessarily available to them, as they do not know of Filipino-identifying ophthalmologists.

## Discussion

Undergoing eye surgery can be an anxiety-inducing undertaking that most participants recognize as necessary, but that some still postpone. This qualitative study was the first to directly explore important factors that shape the decision to obtain eye surgery focused on the cultural lens of Filipino-Americans, a large U.S. minority group. Interviews were conducted with a sample of participants who mostly had good clinical outcomes after surgery. Most participants identified the significance of community relationships in deciding to undergo eye surgery. Although most had an overall positive experience, several participants identified stressors and worries that were not always readily shared with their provider. Some attributed this hesitancy to the cultural concept of “hiya,” which encapsulates the embarrassment in asking clarifying questions and the shame or disdain in questioning the authority of the doctor. This hesitancy may have also been due to the lack of interpreter use which created language barriers. Feelings of vulnerability arising from the inability to talk and express emotions were alleviated with religious practices such as prayers and faith groups. Our research revealed a complex interplay between autonomy, “hiya,” and self-efficacy. By delving deeply into how the Filipino-American population has experienced their eye surgery journey, this study hopes to not present a complete understanding of one culture but rather to provide an overall understanding that patients of diverse cultures and belief systems may perceive health and illness differently through a cultural sieve and therefore respond differently to diseases, symptoms, and treatments.

Our findings highlighted several paths for improvements in developing a culturally tailored ophthalmologic care surgery experience for Filipino-Americans and possibly other ethnic minorities and immigrant populations. When making decisions about their eye surgery, participants reported the integral role of consulting not only with their healthcare providers, but also with families and online resources. Most, if not all, participants highly valued their healthcare providers’ opinions, trusting their words as the experts in their field. Arguably, however, one of the biggest stakeholders in the decision to undergo surgery was the collectivist need to rely on input from participants’ personal support systems [22–24]. The role of the patient’s community cannot be understated, as this component is imperative for the effectiveness of the whole surgical process. One of our participants asked their ophthalmologist, “‘May you wait doctor? May I ask my daughter?’… Then, I told my daughter, and then my daughter approved. So, we… did [the cataract surgery].” In our study, family members, especially those with a health background, were often expected to make decisions or provide input for Filipino-American older adults before pursuing an ophthalmologic procedure; those without any close relatives relied on friends or their faith groups. Hence, for surgeons and providers, it is crucial to consider the patient community and their involvement in the health decision-making process. Providers can incorporate this information during preoperative appointments by asking the patient what they have heard about eye surgery. Many subjects in our study also expressed that researching ahead about eye surgeries helped them make favorable healthcare decisions. Therefore, future eye surgery patients can also conduct their own research not only by looking into online resources, but also by asking their family or friends their thoughts about and experience undergoing eye surgery. Additionally, patients can feel empowered to have the agency to take their time when making their decision and invite friends or family members to be present during informational care appointments. Undertaking a relationship with a surgery patient means that the provider has also inherited their community. Another possible way to consider a patient’s social network is by providing multidisciplinary counseling to patients and their caregivers prior to surgery for a more holistic approach. Evidence shows that patients who receive psychological support in conjunction with surgical care are more likely to have better mental well-being, experience fewer problems following discharge and have a greater self-care ability [25]. Recognizing factors such as culture and community and the role these play in patient perspectives and health decision making upholds delivery of culturally sensitive services as a pillar of high-quality healthcare.

Additionally, several studies have demonstrated that most individuals strongly value their sight and therefore want to be actively involved in every step of their eye surgery care [13, 26–28]. Our data suggest that many of our participants hoped to ask clarifying questions, voice concerns, and define outcome expectations with their provider. However, some felt it was not appropriate to engage in these conversations in the clinic and did not want to be seen as questioning the expertise of the provider. This was also found in other studies [26, 29, 30]. Inherent power dynamics between physicians and patients may act as a deterrent to more open dialogue, especially since this can be intensified by the cultural phenomenon of “hiya,” as mentioned and described by participants themselves. Previous studies have described “hiya” as a social filter towards physicians, in which patients choose not to complain of their pain unless explicitly asked to avoid possibly offending the physician [22]. In our study, “hiya” was exemplified as patients refrained from asking questions from the physician because they feared that they were challenging a person of authority. Although “hiya” can be a virtue that exemplifies the collectivistic Filipino culture, it is important that providers acknowledge its possible presence to ensure that emphasis is placed on adequate probing questions to encourage full expression of their patient’s medical concerns [31]. This will ensure well-informed and patient-centered preoperative conversations, which are key for patients having higher levels of satisfaction with their healthcare [32]. This is especially important for patients receiving more high-risk ophthalmologic care. One of our participants who underwent surgery for their retinal disease experienced an adverse outcome in which they expressed regret about their decision to pursue eye surgery. Moreover, more open physician–patient communication about expectations and outcomes may have prevented feelings of disappointment and confusion.

Additionally, people with minoritized identities have ongoing mistrust toward medical institutions [33, 34]. National conversations have made efforts toward system change and creating a more trustworthy medical field rather than fixing individual mistrust [35]. It is important, then, to recognize when a patient may be hesitant to ask questions, especially when building trust in a racially discordant physician–patient relationship. Attunement toward the patient’s nonverbal communication is one opportunity. Another method to strengthen a therapeutic relationship is to recognize the need for individualize care depending on the specific needs of the patient [26]. Several partnership-building communication tools have been developed that can be utilized to promote physicians’ awareness of their own biases and racism and to develop physicians’ rapport with patients from different cultural backgrounds. King et al. summarized best practices in addressing patient needs six through the “6-function model” of the medical interview: fostering the relationship, gathering information, providing information, decision making, enabling disease- and treatment-related behavior, and responding to emotions [36]. Additionally, professional medical interpreters shared that patients were more likely to openly communicate with physicians if they showed interest and asked questions about a patient’s country of origin to establish rapport, further suggesting the need for more culturally sensitive training for providers [37].

Another key action taken by providers is to recognize limitations in communication, especially when experiencing language barriers. Our findings demonstrated that although almost half of the participants expressed that having an interpreter or a Tagalog-speaking clinician would have improved their eye surgery experience, only two were offered professional medical interpreters. Others relied on Tagalog-speaking ancillary staff for further clarification of instructions. This inconsistent provision of professional interpreters is concerning because language barriers have been shown to cause patient delay in seeking healthcare services and distrust in medical care. It was found that people with minoritized identities, such as Latinos and Asian Americans, may avoid asking for an interpreter in fear of discrimination. One study showed that among Asian Americans, limited English proficiency and discrimination are associated with psychological distress even when adjusted for sociodemographic variables and immigration-related factors [38]. Another study demonstrated that the presence of an interpreter significantly increased the likelihood of follow-up among non-English-speaking patients who experienced an ophthalmologic emergency [39]. Pre-operatively, ensuring the standardization of interpreter use especially when counseling or consenting for the procedure or surgery can help guide surgical and anesthetic management, therefore saving valuable clinical and operating room time. Additionally, patients are usually awake and able to communicate with the surgical team during most eye surgeries. Although many facilities routinely provide interpreter services in the operating room for patients who require them, this practice is not standard across all institutions performing ophthalmic surgeries. Providing medical interpreters for patients with limited English language proficiency intraoperatively can ensure that safety concerns are addressed. For example, a patient may need to communicate their anxieties to their operating surgeon during surgery, which could necessitate adjustments in management such as the administration of more anxiolytic medication to alleviate the patient's distress. Additionally, if a patient exhibits excessive eye or body movements during surgery, it is crucial to instruct them to refrain from moving to ensure the procedure can be conducted safely. These examples illustrate the importance of communication between patients and surgeons, which becomes more difficult when there are linguistic barriers.

In our study, most of our participants were bilingual and preferred to speak English, which may explain the inconsistent offering of interpreters to the patients by the clinics. Additionally, although seven out of thirteen participants, when asked about their preferred language, requested that their study interviews be conducted in Tagalog, only two of the thirteen participants were explicitly offered interpreters during their eye surgery appointments. It is possible that simply offering the option for a professional interpreter may allow for better communication options. Of note, however, a few participants in our study did express offense about the idea of being offered an interpreter, which may be attributed to the notion that proficiency in English is perceived as being closely tied to one’s social class [41]. The offer of interpreter services may bring forth internalized feelings of shame and otherness, as well as enhance the cultural feelings of *hiya* as mentioned earlier. For example, one participant mentioned that “[an interpreter] wouldn't have made a difference. Maybe for others, who totally need a translator… because, for me, for example myself… I know how to speak English. It’s just that, sometimes when you’re speaking to someone white or whatever… It’s like you get a mental block… You kinda have to translate it in your thoughts first before telling them about it.” Although the participant denied a need for an interpreter, they also mentioned how they cannot fully express themselves in English to the non-Tagalog speaking provider. It is important, then, that bilingual patients who experience these difficulties view interpreter services as a vehicle to fully engage in their care and ultimately feel comfortable accepting interpreter services. A way this can be addressed by the larger Filipino-American community is through the development of public service announcements that encourages community members to ask for an interpreter when undergoing critical surgeries such as eye surgery. The public service announcements would also encourage community members to not feel “hiya” and emphasize that interpreter services can improve clinical outcomes and patient satisfaction. These messages can be conducted via TV programs, radio announcements, or presentations in health fairs and community events. Additionally, consistently offering interpreter services as the healthcare provider and finding ways to normalize the use of interpreter services without placing the onus on the patient to ask for an interpreter can further mitigate negative healthcare implications [40]. Providers, for example, can simply offer interpreter services to every patient, especially in a hospital or clinical setting with known linguistic diversity. Including a normalizing sentence when introducing the topic of interpreter services such as “we offer interpreter services to everyone” may additionally diminish feelings of shame. This strikes a balance between respecting the patient’s autonomy and ensuring their well-being, which can enhance their satisfaction with care. However, interpreter services are not be sufficient to alleviate the challenges that come with language barriers and other avenues may need to be explored.

While it is important to address the possible stigma that exists with the use of interpreter services, efforts should be focused on increasing the availability of bilingual providers in specialty care, which has been shown to be more effective in navigating the nuances and challenges of language for delivering optimal care in an ever increasingly diverse U.S. population [42]. Bilingual providers allow patients to speak both English and Tagalog in their preferred language, further increasing patient satisfaction with care and improving outcomes. This was highlighted when a participant stated by speaking in the “Tagalog language, it becomes more intimate in terms of your relationship, and the frankness and the honesty of what is needed. The doctor can calm you down, and you can talk also in Tagalog.” One way this could be achieved is by focusing on further diversifying the ophthalmologic workforce. Racially concordant relationships were shown to be therapeutic, rejoiced, and sought by our study participants. Additionally, it has been shown that a diverse workforce is more likely to be able to speak another language, which may address communication errors. However, racial concordance does not mean linguistic concordance, and it is important that medical providers also have adequate medical language proficiency. Efforts to increase support for language learning and boost the intermediate language proficiency of trainees and providers during professional training can ensure that language proficiency standards are met. These professional trainings, especially when provided by organizations, come with lessons on the culture of the population they are serving, further bolstering culturally sensitive care. Although improved outcomes of linguistically and culturally concordant care [43] are well established, the preference for Filipino ophthalmologists as participants expressed in our study may not be accessible due to the lack of diversity of the ophthalmologic workforce, as nationwide underrepresented minority groups constitute only 7.2% of practicing ophthalmologists in the United States [44, 45]. This may continue to persist, as only one medical school considers Filipinos as underrepresented in medicine [46] despite the continued growth of the ethnic group as the largest Southeast Asian and third largest Asian-American population [7]. The disaggregation of demographic data from larger ethnic groups into more specific subgroups, such as identifying the number of Filipino or Filipino-American ophthalmologists within the larger Asian category, is important for determining patients’ access to culturally concordant care [47]. By designing ophthalmic care for the needs of those who are marginalized, it means designing ophthalmic care for all.

Our study has several limitations. Our sample size is small, with a majority of the subjects interviewed identifying as women. Despite this, themes saturated which suggests consistency in the responses. Additionally, a key limitation of this study was that recruitment data were acquired through purposeful sampling. However, our aim was not to provide results that are generalizable to the population, but rather to provide an in-depth exploration of the eye surgery experience of Filipino-Americans. This focus allows us to explore the eye surgery experience from the perspective of a group that shares a similar cultural and ethnic background, a call to action from several papers [13, 44, 45, 48, 49]. Future studies can concentrate on correlating care outcomes and patient experiences, as well as exploring participants in higher risk eye surgeries to determine whether additional factors influence the health decision-making process. Further studies are needed to understand the optimal ways to offer and destigmatize interpreter use among bilingual patients.

This study helps identify some of the factors that influence health decision-making during the receipt of eye surgery and provides data to assist ophthalmologists in serving this often overlooked and aging population in a culturally sensitive and effective manner. How clinicians convey information is crucial; Filipino-American patients can interpret eye surgery expectations through a cultural lens, which may result in lasting impressions about surgical experience and outcomes. Expanding cultural humility training and interpreter services can help patients feel supported.

## Supplementary Information

Supplementary Material 1.

Supplementary Material 2.

## Data Availability

Data is provided within the manuscript or supplementary information files.
