# Supplementary material for: Experiences of older adult Filipino-Americans surrounding eye surgery and factors in health decision-making: a qualitative study
Source: BMC Health Serv Res. 2024 Dec 18;24:1599. doi: 10.1186/s12913-024-12061-1 (PMC11654260; doi:10.1186/s12913-024-12061-1)
Supplement: Supplementary file 1 — Supplementary Material 1. [file 12913_2024_12061_MOESM1_ESM.docx]

**Filipino American Eye Surgery Patient Experiences**

Qualitative Questions Interview Guide–English version

**I. General Eye Care**

1. I’d love to get to know you more. Can you tell me more about your move into the bay area?
2. Tell me about your eye visits.
   1. What are your expectations for your eye visits?
   2. How did you feel during this time?
   3. Where do you get information about eye care and eye diseases?
   4. Where is the most trusted source of information about eye care and eye diseases?
   5. What has made it easier for you to seek out eye care? More difficult?
3. Tell me about your eye care provider.
   1. How did you choose this person?
   2. Tell me about your relationship with your provider.
      1. Some people I talk to feel pleased with the choices available for providers, others not so much. What about you?
   3. When you started to look for a provider, were you pleased with the choices available? Or were you looking for options that you couldn’t find?
4. How would you rate your vision? Choose one.
   1. a) Poor (impairs daily activities)
   2. b) Fair (some vision problems, but able to perform daily activities without difficulty)
   3. c) Good (no vision problems)
5. Do you have any chronic conditions?
   1. *If they do have diabetes/hypertension:* what do you think about getting eye care when you are living with diabetes?
6. *About their eye condition:* How does having these eye conditions affect your day to day life?
   1. How are you managing these current eye conditions?
   2. What challenges have you experienced in caring for these eye conditions?

**II. Eye Care Procedure/Surgery - Participants who went through the procedure**

1. Tell me about your experience before your surgery.
   1. Tell me about any big events during this time.
   2. What eye procedures/surgeries have you gone through? What is this procedure for?
   3. How did you find out that you needed the procedure/surgery?
   4. How did you feel when you learned that you needed a procedure/surgery? What were your initial thoughts when you learned that you needed a procedure/surgery?
   5. Why did you decide to undergo this procedure or surgery?
      1. Who made this decision? Who helped you make this decision?
   6. What results were you expecting from this procedure?
2. Tell me about your experience during this surgery/procedure
   1. How was your experience of undergoing this procedure/surgery?
   2. Who was there?
   3. If you could change things about your surgery, what would you change?
3. Oftentimes when ophthalmologists are doing surgery in the operating room, we do not have the option of having a translator/interpreter either in person or on the phone to speak the patient’s native language.
   1. Did you have a translator/interpreter during your surgery?
   2. *If not:* What was the experience like?
      1. *Note feelings of isolation, loss of agency*
   3. Did you feel like your care would have been better if you had access to a translator/interpreter?
      1. *Note if they say it would have made them feel safer or address needs*
4. What happened after the surgery?
   1. How did you feel?
   2. Were your expectations met?
   3. *(If the respondent notes something wrong with the providers)* Did you ever speak with your physician about what happened? Tell me about that.
   4. What happened in the months after the surgery?
   5. What happened in the years after the surgery?
   6. How did this experience affect your life?
      1. How did this experience affect others in your life?
   7. Looking back on the experience, do you see things about the experience differently than you did at the time? How do you feel like that experience continues to affect you?
5. How well did you feel equipped to take care of your eyes?
6. If you could go back and change things about before, during or after your surgery, what would you change?
7. What would you wish you have known?

**III. Eye Care Procedure/Surgery–Participants who have gone through a procedure/surgery in the past, AND are being advised to go through another procedure now** *(if applicable, proceed to X)*

- How does your past experience affect your decision to get the next procedure?

**IV. COVID-19 and Eye Surgery**

- How has COVID-19 affected your eye care lately? Ability to get health care?
- How has COVID-19 affected your post-surgery care?

**V. Model of Empowerment and Racism**

- Some people in our community have said that racism is a larger problem. Some people have felt like it has impacted them personally. How do you feel like it has impacted you?
  - How do you think it has impacted you in health care? Eye care?
  - *Probing question/comments:* can reference the anti-asian hate right now
- How well did you feel that your eye provider understood you?
  - *Probing question:* did you feel heard?
- How would you feel if you had a Filipino-American physician?
  - *Probing question:* Did you wish that you had an eye provider/doctor of their same background, but were unable to find one?

**VI. Community sources of knowledge/ what is the community sense**

- Within the Filipino community, where, when, and with whom do people talk about your vision?
  - *Probing:* eye care instead of vision
- What advice would you give to other Filipinos who are told that they need an eye surgery?
  - How would you encourage other older Filipino Americans in getting eye surgery?

**VII. Thinking of next steps in facilitators and barriers**

- What are your eye care plans for the future?

**VIII. Concluding Remarks**

- What else should we know about this topic?
- How was the interview for you?

**IX. Post Interview:** *Normalizing the person’s feelings and experience by reminding him or her that it is not unusual for people to have strong feelings arise following this type of interview)*

- “We have covered a lot of territory over the past hour. How are you doing right now?”

Additional Questions (very last thoughts, which may or may not be asked):

**X. Eye Care Procedure/Surgery - Participants who were informed they need a procedure**

1. Tell me about the procedure/surgery that you have been advised to go through.
   1. What eye procedures or surgeries are you/were you being asked to go through? What is this procedure for?
   2. How did you find out that you needed the procedure/surgery?
   3. How did you feel when you learned that you needed a procedure/surgery? What were your initial thoughts when you learned that you needed a procedure/surgery?
   4. What factors were you considering on whether or not you should undergo the procedure?
      1. What is a percent chance that you will decide to take the procedure?
      2. Who is responsible for making this decision?
   5. What results are you expecting from the procedure?
   6. What questions would you like answered about the procedure?
   7. What would help you decide about whether or not to undergo this procedure?

**XI. If they have not seen an eye doctor**

1. Can you share with me why you have not had your eyes examined?
   1. What are some factors that prevent you from going to the eye doctor for an eye exam?
   2. Has your medical doctor (PCP) ever talked to you about getting an eye exam?
   3. Do you have any fears about going to the eye doctor?
   4. Tell me what factors would make you more comfortable with going to the eye doctor?

**Filipino American Eye Surgery Patient Experiences**

Qualitative Questions Interview Guide–Tagalog version

**I. General Eye Care**

1. Gusto po naming mas kilalanin kayo. Maari po ba ninyong ilarawan ng kaunti ang tungkol sa iyong sarili?
2. Kumusta po ang inyong pagpapatingin o pagpapacheck-up sa mata?
   1. Kailan po ang huling beses na kayo ay nagpacheck-up ng mata?
   2. Ano po ang inanahasan ninyo na mangyari sa inyong pagpapacheck up ng mata?
   3. Ano po ang inyong pakiramdam habang kayo ay nagpapacheck-up?
   4. Saan kayo nakakakuha ng impormasyon tungkol sa pag-aalaga sa mata at sa mga sakit sa mata?
   5. Saan po ninyo nahahanap ang mga pinka-pinag-kakatiwalaan ninyong impormasyon tungkol sa pagaalaga ng mata at mga sakit sa mata?
   6. Ano po ang mga bagay na nakatulong upang mapadali ang inyong paghahanap ng mga dapat gawin upang pangalagaan ang mata? Ano ang mga bagay na mas nagpapahirap dito?
3. Kumusta po ang inyong doktor sa mata?
   1. Paano ninyo napili ang doktor na ito?
   2. Kumusta po ang inyong relasyon sa inyong doktor sa mata?
      1. Ang iba naming mga nakausap ay kontento sa mga napagpipilian nilang mga doktor sa mata, pero ang iibang mga tao ay medyo hindi gaanong kontento sa kanilang mga pinagpipilian. Kayo po, ano ang inyong opinyon?
   3. Noong kayo ay nagumpisa na maghanap sa ng mga doktor sa mata, kontento po ba kayo sa mga napagpipilian? Mayroon po ba kayong ibang mga hinahanap?
4. Kumusta po ang inyong paningin ngayon? Pumili po kayo sa isa sa mga sumusunod
   1. a) Hindi na maayos (sagabal sa mga pang-araw araw na gawain)
   2. b) Medyo maayos (medyo merong problema sa paningin, pero maayos na nakakagawa ng mga pang-araw araw na gawain)
   3. c) Maayos (walang problema sa paningin)
5. Maryoon po na kayong mga pang-matagalan na sakit o karamdaman?
   1. *If they do have diabetes/hypertension:* Kumusta po ang inyong pagaalaga at pag-papacheck up ng mata ng may sakit ng ____
6. *About their eye condition:*Paano po nakakaapekto ang kondisyon ng inyong mga mata sa inyong pang-araw araw na pamumuhay?
   1. Paano po ninyo pina-pa-maha-laan ang inyong mga kondisyon sa mata?
   2. Ano pong mga hamon ang iyong nararanasan sa pag-aalaga sa inyong mga kondisyon sa mata?

**II. Eye Care Procedure/Surgery - Participants who went through the procedure**

1. Kumusta po ang inyong karanasan bago ang inyong operasyon?
   1. Sabihin po ninyo sa aming ang tungkol sa anumang mga malalaking pangyayari na inyong natatandaan bago ang operasyon.
   2. Ano po ang mga operasyon sa mata na inyo nang naranasan? Para saan po ang operasyon na ito?
   3. Paano po ninyo nalaman na kailangan niyo pala ang operasyon na ito?
   4. Ano po ang inyong naramandaman noong nalaman ninyo na kailangan niyo ng operasyon? Ano po ang inyong mga bagay na unang pumasok sa inyong isip noong nalaman ninyo na kailangan ninyo ng operasyon sa mata?
   5. Bakit po ninyo pinili na magpa opera ng mata?
      1. Sino po ang nagpasya na magpaopera kayo?
   6. Ano po ang mga resulta na inyong inaasan mula sa operasyon na ito?
2. Kumusta po ang inyong karanasan sa pagganap ng operasyon?
   1. Kumusta po ang inyong karanasan sa mismong oras operasyon?
   2. Sino po yung nandoon na kasama ninyo?
   3. Kung maari kayong may baguhin sa inyong operasyon, ano po ang inyong pipiliin na baguhin?
3. Kadalasan po, kapag ang ophthalmologist o doktor sa mata ay nagsasagawa ng operasyon, walang opsyon na magkaroon ng translator o interpreter/tagapagsalin (sa personal o sa telepono) na maari ninyong kausapin gamit ang inyong sariling wika.
   1. Nagkaroon po ba kayo ng translator/interpreter/tagapagsalin habang isinasagawa ang inyong operasyon?
   2. *If not:* Kumusta po ang inyong karanasan?
      1. *Note feelings of isolation, loss of agency*
   3. Sa inyong opinyon, mas mapapabuti po ba ang inyong karanasan kung nagkaroon kayo ng translator/interpreter/tagapagsalin?
      1. *Note if they say it would have made them feel safer or address needs*
4. Ano po ang nangyari pagkatapos ng operasyon?
   1. Ano po ang naramdaman ninyo?
   2. Nakamit ba ang resulta na inyong inaasahan?
   3. (If the respondent notes something wrong with the providers) Kinausap po ba ninyo ang inyong doktor tungkol dito? Kumusta po yung usapan na ito?
   4. Ano po ang nangyari sa mga susunod na buwan pagkatapos ng inyong operasyon?
   5. Ano po ang nangyari sa mga susunod na taon pagkatapos ng inyong operasyon?
   6. Ano po ang epekto nitong operasyon sa inyong buhay?
      1. Ano po ang epekto nito sa mga ibang tao sa inyong buhay?
   7. Sa inyo pag balik tanaw po ninyo sa karanasan na ito, may pagkakaiba po ba sa inyong pananaw ngayon kumpara sa inyong pananaw sa sitwasyon noong nakaraan? Paano po nito kayo patuloy na naapektuhan?
5. Gaano po ninyo naiintindihan kung ano ang mga kailangan gawin upang alagaan ang inyong mata? Gaano po kayo kahanda na alagaan ang inyong mga mata?
6. Kung bibigyan po kayo ng pagkakataon na balikan ang mga oras bago, habang, o pagkatapos ng operasyon, mayroon po ba kayong pipiliin na baguhin sa anumang parte nito?
7. Ano po ang mga ninais ninyo na kaalaman noong bago sana kayo nagpaopera?

**III. Eye Care Procedure/Surgery–Participants who have gone through a procedure/surgery in the past, AND are being advised to go through another procedure now** *(if applicable, proceed to X)*

- Paano po nakakaapekto ang inyong nakaraan na karanasan sa inyong desisyon sa inyong susunod na pagpapaopera?

**IV. COVID-19 and Eye Surgery**

- Paano po nakaapekto ang COVID-19 sa inyong karaniwang pag-aalaga sa mata?
- Paano po nakaapekto ang COVID-19 sa inyong pag-aalaga sa mata pagkatapos ng operasyon?

**V. Model of Empowerment and Racism**

- Iilang mga tao sa ating komunidad ay nagsasabi na ang racism o rasismo ay isang mas malaking problema at mayroong iba na personal na apektuhan nito. Paano po ito nakakaapekto sa inyo ngayon?
  - Paano po ito nakaapekto sa inyong pagaalaga ng kalusugan? Sa pag-aalaga sa inyong mga mata?
  - *Probing question/comments:* can reference the anti-asian hate o karahasan na nararanasan ng ating komunidad ngayon
- Sa inyong opinyon, gaano kahusay kayo naiintindihan ng inyong doktor sa mata?
  - *Probing question:* Pinapakinggan ka ba ng iyong doktor?
- Ano po ang inyong mararamdaman kung kayo ay magkakaroon ng doktor na Filipino-American/ Pilipino-Amerikano?
  - *Probing question:* Hihilingin po ba ninyo na mayroon kayong doktor sa mata na nanggagaling mula sa kapareho ninyong kultura o background? Nahihirapan po ba kayong hanapin ito?

**VI. Community sources of knowledge/ what is the community sense**

- Sa inyo pong komunidad na Pilipino, saan, kailan, at sino ang mga kinakausap ninyo tungkol sa inyong paningin sa mata?
  - *Probing:* pag-aalaga sa mata (instead of paningin sa mata)
- Ano po ang magiging payo ninyo sa mga ibang Pilipino na nasabihan na kailangan nila ng operasyon sa mata?
  - Paano po ninyo mapapalakas ang loob ng mga ibang Pilipino na kailangan na magpaopera ng mata?

**VII. Thinking of next steps in facilitators and barriers**

- Ano po ang inyong mga plano sa pag-aalaga ng mata sa hinaharap?

**VIII. Concluding Remarks**

- Ano po ang mga iba pang mga bagay na gusto ninyong sabihin saamin tungkol sa tema na ito?
- Kumusta po itong panayam namin sa inyo?

**IX. Post Interview:** *Normalizing the person’s feelings and experience by reminding him or her that it is not unusual for people to have strong feelings arise following this type of interview)*

- “We have covered a lot of territory over the past hour. How are you doing right now?”
- “Marami na po tayong napagusapan sa nakalipas na isang oras. Kumusta po ang inyong kalagayan ngayon?”

Additional Questions (very last thoughts, which may or may not be asked):

**X. Eye Care Procedure/Surgery - Participants who were informed they need a procedure**

1. Sabihin niyo po saamin ang tungkol sa operasyon na napayuhan kayo na inyong kailangan.
   1. Ano po ang mga operasyon sa mga mata na pinayuhan kayo na inyong kailangan? Para saan po ang operasyon na ito?
   2. Paano po ninyo nalaman na kailangan niyo ng operasyon?
   3. Ano po ang inyong naramandaman noong nalaman ninyo na kailangan niyo ng operasyon? Ano po ang inyong mga bagay na unang pumasok sa inyong isip noong nalaman ninyo na kailangan ninyo ng operasyon sa mata?
   4. Ano pong mga bagay ang inyong kinokonsidera habang pinagiisipan inyo kung kayo ay magpapaopera o hindi?
      1. Ano pong porsyento ng pagkakataon na pipiliin ninyong magpaopera ng mata?
      2. Sino po ang magiging responsable sa paggawa ng desisyoon na ito?
   5. Ano po ang mga resulta na inyong inaasan mula sa operasyon na ito?
   6. Ano po ang mga tanong na gusto ninyong masagot tungkol sa operasyon?
   7. Ano po ang makakatulong sa inyo na magpasya kung tatanggapin ninyo o hindi ang operasyon na ito?

**XI. If they have not seen an eye doctor**

1. Maari po ba ninyong sabihin saamin kung bakit napili ninyo na hindi muna magpa check up ng mata?
   1. Ano po ang mga bagay na humahadlang sa inyong pagpunta sa doktor para ipatingin ang inyong mga mata?
   2. Nasabihan na po ba kayo ng inyong PCP o primary na doktor na kailangan ninyong magpatingin ng mata?
   3. Mayroon po ba kayong mga pangangamba sa inyong pagpunta sa doktor sa mata?
   4. Ano pong mga bagay na magiging komportable ka sa pagpunta sa doktor sa mata?
