# Supplementary material for: Experiences of older adult Filipino-Americans surrounding eye surgery and factors in health decision-making: a qualitative study
Source: BMC Health Serv Res. 2024 Dec 18;24:1599. doi: 10.1186/s12913-024-12061-1 (PMC11654260; doi:10.1186/s12913-024-12061-1)
Supplement: Supplementary file 2 — Supplementary Material 2. [file 12913_2024_12061_MOESM2_ESM.docx]

**Filipino American Eye Surgery Patient Experiences**

Qualitative Research Screening and Eligibility Questions

*I’m so glad that you are interested in taking part in our study. For the first part of our study, we have to ask you a few questions to make sure you are eligible for the study. Do you prefer to have this interview in English or Tagalog?*

**Screening & Eligibility Questions**

1. Do you identify as Filipino-American?
2. Where do you currently reside?
   1. What county?
3. How old are you?
4. What eye surgery did you get, where did this happen, and when did this happen? (cataract, glaucoma, and retinal)
5. When are you available for an interview?
